# Triptolide Administration Alters Immune Responses to Mitigate Insulin Resistance in Obese States

**DOI:** 10.3390/biom14040395

**Published:** 2024-03-25

**Authors:** Lyudmila Grodsky, Mickey Wilson, Thirumurugan Rathinasabapathy, Slavko Komarnytsky

**Affiliations:** 1 Plants for Human Health Institute, North Carolina State University, 600 Laureate Way, Kannapolis, NC 28081, USA; lyudmila_grodsky@med.unc.edu (L.G.); mlwilso8@ncsu.edu (M.W.); trathin@ncsu.edu (T.R.); 2 Department of Post-Baccalaureate Studies, University of North Carolina at Charlotte, 9201 University City Blvd, Charlotte, NC 28223, USA; 3 School of Medicine, University of North Carolina at Chapel Hill, 150 Medical Drive, Chapel Hill, NC 27514, USA; 4 Department of Food, Bioprocessing, and Nutrition Sciences, North Carolina State University, 400 Dan Allen Drive, Raleigh, NC 27695, USA

**Keywords:** metabolic syndrome, immunometabolism, metabolic deregulations, metabolic disorders, energy metabolism, diabetes mellitus, mitochondria, molecular targets, biomarkers

## Abstract

Individuals who are overweight or obese are at increased risk of developing prediabetes and type 2 diabetes, yet the direct molecular mechanisms that connect diabetes to obesity are not clear. Chronic, sustained inflammation is considered a strong risk factor in these interactions, directed in part by the short-lived gene expression programs encoding for cytokines and pro-inflammatory mediators. In this study, we show that triptolide administration in the C57BL/6 diet-induced obese mice at up to 10 μg/kg/day for 10 weeks attenuated the development of insulin resistance and diabetes, but not obesity, in these animals. Significant reductions in adipose tissue inflammation and improved insulin sensitivity were observed in the absence of changes in food intake, body weight, body composition, or energy expenditure. Analysis of the core cluster of biomarkers that drives pro-inflammatory responses in the metabolic tissues suggested TNF-α as a critical point that affected the co-development of inflammation and insulin resistance, but also pointed to the putatively protective roles of increased COX-2 and IL-17A signaling in the mediation of these pathophysiological states. Our results show that reduction of diet-induced inflammation confers partial protection against insulin resistance, but not obesity, and suggest the possibility of achieving overweight phenotypes that are accompanied by minimal insulin resistance if inflammation is controlled.

## 1. Introduction

Obesity is a complex metabolic dysfunction driven in part by the elevated calorie surplus and energy density of modern diets. As public efforts to promote healthy eating and active living in a variety of settings around the world have been of limited success, obesity rates continue to increase [1]. Obesity also raises the risk of debilitating morbidity and mortality through a variety of cardiometabolic complications that include type 2 diabetes mellitus, hypertension, dyslipidemia, non-alcoholic fatty liver, atherosclerosis, ischemic cardiovascular disease, and certain cancers [2]. The prevalence of these pathologies, however, trails the rise of obesity with an average delay of 10–15 years as shown for diabetes [3], and the progressive damage from sustained hyperglycemia and impaired insulin action in the target tissues is suspected to be responsible for this observation [4]. Chronic inflammation and immune dysregulation are believed to play a major role in the onset and progression of metabolic disorders [5], yet a clear link between the development of obesity and diabetes is lacking. This is further complicated by the existence of obesity-prone as well as obesity-resistant phenotypes [6], as well as metabolically healthy overweight individuals that seem to maintain normal glucose, blood pressure, and lipid levels [7]. Metabolic inflexibility in the sensing and processing of various nutrient substrates is also suspected to contribute to these outcomes [8]. Critically, modern anti-diabetic drugs that target glucose control (metformin, GLP-1 analogs such as liraglutide or taspoglutide, and SGLT-2 inhibitors such as dapagliflozin or empagliflozin) somewhat mitigate cardiovascular risk factors but fail to resolve many inflammation-associated diabetes outcomes such as non-alcoholic fatty liver and retinopathy.

A close connection between obesity, inflammation, and insulin resistance is further exemplified by the fact that a gradual weight loss of 5–15% of the original body weight over 3–10 months is sufficient to improve β-cell function and insulin sensitivity in all metabolically active tissues [9]. Expansion of fat depots to accommodate excess lipid accumulation requires upregulation of inflammatory mechanisms that allow for perfusion and remodeling of the adipose tissue. This is generally achieved by the activation of transitory and resident macrophages, and the secretion of immune mediators or humoral factors that contribute to decreased insulin signaling and increased insulin resistance [10]. At the core of this cluster lies tumor necrosis factor alpha (TNF-α), which activates JNK and IKKβ signaling, sustains adipose inflammation, and promotes insulin resistance [11]. This pro-inflammatory response mimics inflammation-induced insulin resistance, also observed in sepsis [12], burn injury [13], and normal pregnancy [14]. Moreover, early attempts to target insulin resistance with salicylic acid [15], salicilates [16], aspirin [17], or antirheumatic drugs [18] showed some promise in ameliorating peripheral insulin resistance.

The C57BL/6J mouse model of diet-induced polygenic obesity (DIO) is a useful tool to unravel the complexity of developmental obesity and associated insulin resistance [19]. In this study, we used pharmacological supplementation with triptolide, a potent anti-inflammatory diterpenoid epoxide that induces rapid proteasome-dependent degradation of the RNA polymerase II [20] and the nuclear factor IκBα [21], to effectively reduce inflammation in body tissues and study the development of diet-induced obesity and insulin resistance when the inflammatory component was suppressed. We quantified changes in food intake, body weight, body composition, energy expenditure, glucose tolerance, insulin sensitivity, and gene networks responsible for the production of the immediate mediators of the inflammatory response.

## 2. Materials and Methods

### 2.1. Reagents and Diets

All chemical reagents and solvents, including triptolide, were purchased from Sigma (St. Louis, MO, USA). Triptolide was formulated both into the 10 kcal % from fat diet D12450J (low fat or LFD, 3.85 kcal/g) and the 60 kcal % from fat diet D12492 (high fat or HFD, 5.24 kcal/g) at 0.1 μg/g food by Research Diets (New Brunswick, NJ, USA). The triptolide dose was selected based on the results of our previous pilot studies with oral administration of triptolide at 50 μg/kg for 9 weeks [22]. The formulations were developed based on our previously recorded C57BL/6J daily food intake of 2.4–3.0 g/mouse/d [23]. Using this data, we established that animals consumed up to 10 μg/kg/d triptolide for the duration of the study. The human equivalent dose was estimated at 0.8 μg/kg/d using a factor of 12.3 [24]. All animal diets were kept at −20 °C to ensure long-term storage and stability.

### 2.2. Animal Study

Male, 6-week-old C57BL/6J mice were purchased from the Jackson Laboratory (Bar Harbor, ME, USA) and housed four animals per cage under controlled temperature (24 ± 2 °C) and light (12 h light–dark cycle, lights on at 7:00 am). Upon arrival, animals were allowed to adapt to new conditions for 7 days, and handling of the animals was performed daily to reduce the stress of physical manipulation. Mice were then randomized into 2 groups (*n* = 16) with ad libitum access to the LFD (3.85 kcal/g) or HFD (5.24 kcal/g) diet for 6 weeks. Next, animals were further randomized into 4 groups (*n* = 8), including LFD (lean), LFDT diet supplemented with triptolide at 0.1 μg/g food, HFD (obese), and HFDT diet supplemented with triptolide at 0.1 μg/g food. The study was performed as a parallel arm with shared LFD and HFD controls [25]. All animal experimental procedures were carried out at the DHMRI Center of Laboratory Animal Sciences, an AAALAC-accredited facility, and approved by the NC Research Campus IACUC.

### 2.3. Animal Body Weight, Composition, and Endurance

Animal weight and food intake (accounting for spillage) were recorded twice weekly for the duration of the study. Calorie intake and feed efficiency (grams of weight gain per gram of food intake) were calculated weekly and at the end of the treatment period. Body composition analysis was performed on unanesthetized mice using EchoMRI (Echo Medical Systems, Houston, TX, USA) during study week 15. An endurance run was performed at the end of the study using the Exer3/6 treadmill (Columbus Instruments, Columbus, OH, USA) set at 0° inclination, 1 Hz frequency, and 0.34 mA intensity. Following 2 min of acclimation, the initial speed was set to 16 cm/s and increased in increments of 2 cm/s every 2 min, until a maximum speed of 24 cm/s was reached.

### 2.4. Oral Glucose Tolerance and Insulin Tolerance Tests

For the oral glucose tolerance test (OGTT), mice were fasted overnight (16 h) and received oral gavage of D-glucose (1.5 g/kg body weight). For the insulin tolerance test (ITT), mice were fasted for 4 h and received intraperitoneal injections of insulin (0.75 U/kg body weight). Blood glucose concentrations were measured at 0, 30, 60, and 120 min (OGTT) and 0, 20, 40, 80, and 120 min (ITT) after challenge in blood samples obtained from the tail-tip bleedings using a Lifescan glucometer (Johnson and Johnson, New Brunswick, NJ, USA). Both measures were taken during study week 16.

### 2.5. Animal Energy Expenditure and Metabolism

Energy expenditure was quantified by indirect calorimetry on an open-circuit LabMaster Metabolism Research Platform (TSE Systems, Bad Homburg, Germany) provided by the UNC Nutrition Obesity Research Center (Kannapolis, NC, USA) during study week 15. Rates of oxygen consumption (VO_2_) and carbon dioxide production (VCO_2_) were measured, relative to a reference cage, during 1 min every 12 min for 48 h, of which the last 24 h were used. Respiratory exchange ratio (RER) was defined as VCO_2_/VO_2_, and energy expenditure (EE) was calculated using the equation [3.815 + (1.232 × RER)] × VO_2_. Carbohydrate and lipid oxidation rates were calculated using the table of nonprotein respiratory quotients [26]. Activity was simultaneously measured with the ActiMot system (TSE) by counting infrared beam breaks in horizontal (x and y directions, running) and vertical (z direction, rearing) planes.

Fresh fecal pellets were collected during cage observations and weighed (*n* = 20). Pellets were dried, powdered, and then 100 mg were suspended in 0.5 mL of PBS. Total fecal lipids were extracted by partitioning into 0.5 mL of chloroform/methanol (2:1), centrifuging at 1000× *g* for 10 min, and evaporating solvents from the lipid phase. The lipid content in feces was calculated as a percent of the total feces weight.

### 2.6. RNA Extraction, Purification, and cDNA Synthesis

The total RNA was isolated from the metabolically active tissues using TRIzol reagent (Life Technologies, Carlsbad, CA, USA) following the manufacturer’s instructions. RNA was quantified using the Biotek SynergyH1/Take 3 plate (Agilent, Santa Clara, CA, USA). The cDNAs were synthesized on ABI GeneAMP 9700 using the high-capacity cDNA Reverse Transcription kit and 2 µg of RNA (Life Technologies).

### 2.7. Quantitative PCR Analysis

The resulting cDNA was amplified by real-time quantitative PCR using the SYBR green PCR master mix (Life Technologies). To avoid interference due to genomic DNA contamination, only intron-overlapping primers were selected using Primer Express software, version 2.0 (Applied Biosystems, Foster City, CA, USA). The following primers were used to capture the core cluster of the short-lived inflammatory gene expression networks, including TNF-α, forward primer: 5′-GTT CTA TGG CCC AGA CCC TCA CA-3′, reverse primer: 5′-TAC CAG GGT TTG AGC TCA GC-3′; IL-1β, forward primer: 5′-CAA CCA ACA AGT GAT ATT CTC CAT G-3′, reverse primer: 5′-GAT CCA CAC TCT CCA GCT GCA-3′; IL-6, forward primer: 5′-TAG TCC TTC CTA CCC CAA TTT CC-3′, reverse primer: 5′-TTG GTC CTT AGC CAC TCC TTC-3′; IL-17A, forward primer: 5′-ATC TGG TCC TAC ACG AAG CC-3′, reverse primer: 5′-GTC CCG GAC TTC AAG ACC C-3′; IL-18, forward primer: 5′-AGG ACA AAG AAA GCC GCC TC-3′, reverse primer 5′-TCA TTT CCT TGA AGT TGA CGC AAG AGT-3′; COX-2, forward primer: 5′-TGG TGC CTG GTC TGA TGA TG-3′, reverse primer: 5′-GTG GTA ACC GCT CAG GTG TTG-3′; iNOS, forward primer: 5′-CCC TCC TGA TCT TGT GTT GGA-3′, reverse primer: 5′-TCA ACC CGA GCT CCT GGA A-3′; and β-actin as a housekeeping gene, forward primer: 5′-AAC CGT GAA AAG ATG ACC CAG AT-3′, reverse primer: 5′-CAC AGC CTG GAT GGC TAC GT-3′.

Quantitative PCR (qPCR) amplifications were performed on an ABI 7500 Fast real-time PCR (Life Technologies) using 1 cycle at 50 °C for 2 min and 1 cycle at 95 °C for 10 min, followed by 40 cycles of 15 s at 95 °C and 1 min at 60 °C. The dissociation curve was completed with 1 cycle of 1 min at 95 °C, 30 s at 55 °C, and 30 s at 95 °C. mRNA expression was analyzed using the ΔΔCT method and normalized with respect to the expression of the β-actin housekeeping gene. Amplification of specific transcripts was confirmed by obtaining melting curve profiles.

### 2.8. Cell Culture

The mouse macrophage cell line RAW 264.7 (ATCC code TIB-71) was maintained in Dulbecco’s modified Eagle’s medium (DMEM, Life Technologies), supplemented with 10% fetal bovine serum (Life Technologies), 100 IU/mL of penicillin, and 100 µg/mL of streptomycin (Fisher Scientific, Pittsburg, PA, USA) at a density not exceeding 5 × 10^5^ cells/mL. Cells were routinely passaged every 3–4 days in Nunc cell culture dishes (Nalge Nunc International, Rochester, NY, USA) maintained at 37 °C and 5% CO_2_ in a humidified Thermo Forma Series II incubator (Fisher Scientific). Cell viability and dose range determination studies were performed using the MTT assay as described. IC_50_ was calculated by a non-linear regression using 5–6 serial dilutions of the test samples.

### 2.9. Analytical Chemistry and HPLC

Sequential extracts of peeled (bark removed) *Tripterygium wilfordii* Hook F roots were analyzed for triptolide content using a Shimadzu Prominence LC-2030C system equipped with an Ultra C18 column (250 mm × 4.6 mm, 5 μm dp, Restek, Bellefonte, PA, USA) and a Restek Ultra C18 guard column (10 mm × 2.1 mm, 5 μm dp). The fractions were centrifuged at 3000 rpm for 5 min and evaporated to dryness using Rotovapor R210 (Büchi, New Castle, DE, USA). The residues were dissolved in 1 mL of methanol, filtered through a syringe with a 0.45 μm PFTE membrane, and 20 μL were subjected to the HPLC analysis of triptolide content at 219 nm.

### 2.10. Statistical Analysis

Statistical analyses were performed using Prism 9.0 (GraphPad Software, San Diego, CA, USA). Data were analyzed by one-way or two-way ANOVA with treatment as a factor. Post hoc analyses of differences between individual experimental groups were made using Dunnett’s multiple comparison tests. The significance was set at *p* < 0.05. Values were reported as means ± SEM.

## 3. Results

### 3.1. Triptolide Administration Improves Insulin Resistance without Affecting Obesity

**Figure 1:**
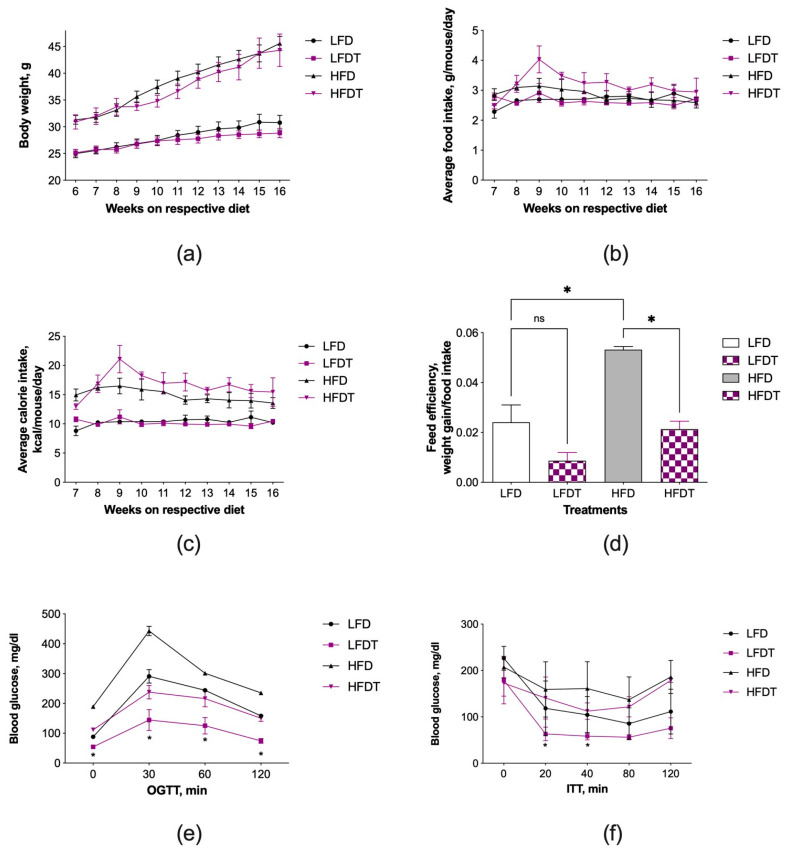
Effect of triptolide administration in lean (LFD) and obese (HFD) C57BL/6J mice. (**a**) Body weight; (**b**) food intake; (**c**) calorie intake; (**d**) feed efficiency; (**e**) oral glucose tolerance (OGTT); and (**f**) insulin tolerance as a measure of insulin resistance (ITT). Following a 6-week randomization to LFD or HFD diets, half of the animals received triptolide in food (0.1 μg/g food, LFDT or HFDT) for 10 weeks (study weeks 7–16). Results were expressed as means ± SEM (*n* = 8). Data were analyzed by the two-way repeated measures ANOVA with time and treatment as independent variables and the Tukey’s multiple comparison test (* *p* < 0.05 vs. the respective controls; ns, non-significant).

At the beginning of the study, animals were randomized to LFD or HFD diets for 6 weeks to allow for the onset of obesity in animals consuming HFD, resulting in increased mean body weights of 30.9 g vs. 25.1 g for lean controls (Figure 1).

At this point, triptolide administration (0.1 μg/g food) was initiated and continued for an additional 10 weeks (study weeks 7–16). At the end of the study, no significant differences in body weights were noted between LFD and LFDT mice (30.7 g vs. 28.8 g) or HFD and HFDT mice (45.6 g vs. 44.3 g) (Figure 1a). Animal food intake was recorded in the range of 2.5–3.2 g/mouse/d, similar to our previous observations [23]. Average food and calorie intake was slightly elevated in HFDT animals (Figure 1b,c), resulting in decreased feed efficiencies that reached significance in HFDT mice (Figure 1d).

Baseline blood glucose levels were measured on week 15 and showed an expected significant increase associated with the HFD diet (189 mg/dL vs. 88 mg/dL in the LFD controls). HFD animals also showed impaired oral glucose tolerance compared to the LFD controls, as the area under curve (AUC) analysis indicated a 45% increase in whole glucose excursion after OGTT (Figure 1e). Triptolide administration decreased blood glucose AUC by 49% in the lean LFDT mice and by 38% in the obese HFDT mice when compared to the respective controls, and the effects were significant across all timepoints (Figure 1e). At the same time, insulin sensitivity was enhanced in animals administered triptolide, irrespective of whether they were fed a LFD or HFD diet. AUC analysis showed that insulin resistance declined by 36% in LFDT mice and 15% in HFDT mice versus the respective controls and reached significance at 20 and 40 min after the ITT challenge (Figure 1f).

### 3.2. Changes in Body Composition and Fecal Lipids

**Figure 2:**
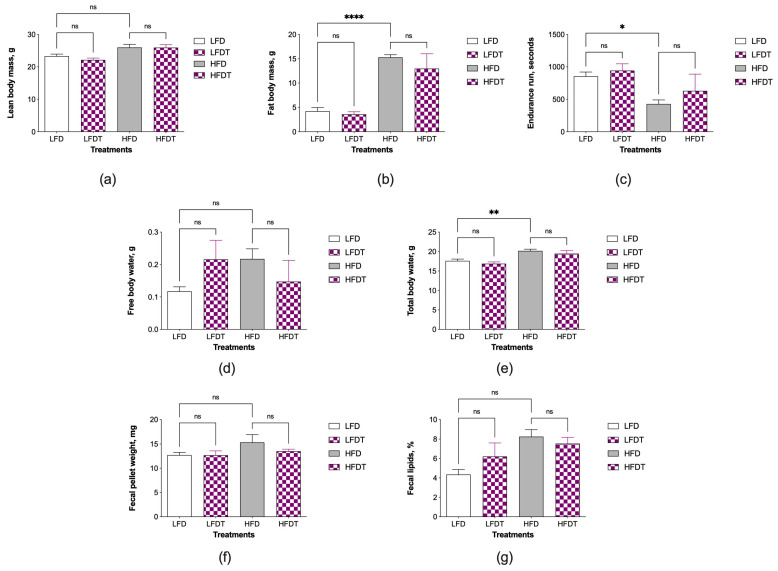
Effect of triptolide administration on parameters associated with body composition and fecal lipids in C57BL/6J mice. (**a**) Lean body mass; (**b**) fat body mass; (**c**) endurance run time; (**d**) free body water; (**e**) total body water; (**f**) fecal pellet weight; and (**g**) total fecal lipids. Results were expressed as means ± SEM (*n* = 8). Data were analyzed by the one-way ANOVA and Tukey’s multiple comparison test (* *p* < 0.05, ** *p* < 0.01, **** *p* < 0.0001 vs. the respective controls; ns, non-significant).

Body composition of the HFD controls showed the appropriate changes in lean body mass (Figure 2a), fat body mass *p* < 0.0001 (Figure 2b), running endurance *p* < 0.05 (Figure 2c), free body water (Figure 2d), total body water *p* < 0.01 (Figure 2e), fecal pellet weight (Figure 2f), and total fecal lipids (Figure 2g). Administration of triptolide was not associated with significant changes in any of these parameters, suggesting that triptolide at 0.1 μg/g food had no measurable effects on the development of obesity in HFD mice. There was a trend for a marginally decreased fat body mass in both LFDT and HFDT treatments, but it has not reached significance (Figure 2b).

### 3.3. Changes in Energy Metabolism and Activity

**Figure 3:**
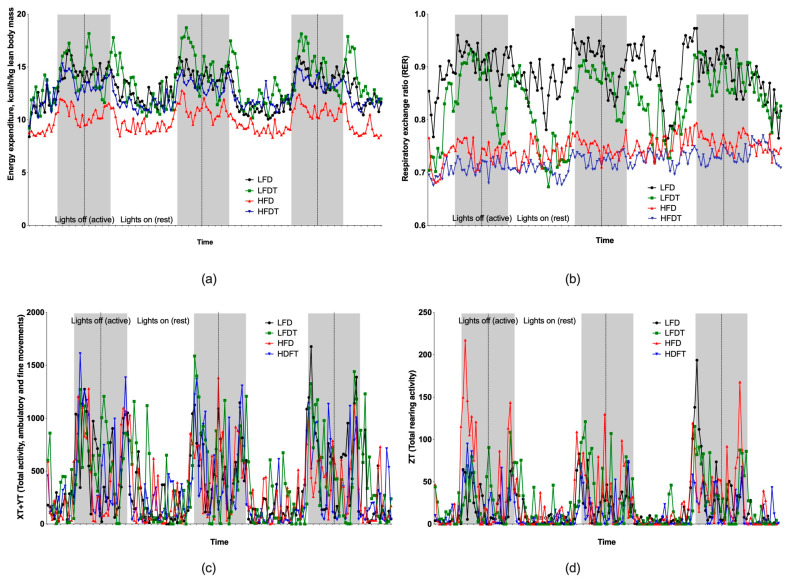
Effect of triptolide administration on energy expenditure and activity of C57BL/6J mice. (**a**) Energy expenditure normalized to lean body mass; (**b**) respiratory exchange ratio; (**c**) total movement activity along the XY axes; and (**d**) total rearing activity along the Z axis.

At the end of the study, animals were monitored in the metabolic TSE chambers for three full days, which included a normal 12 h diurnal cycle with lights off (active phase) and lights on (inactive phase). LFDT animals showed on average two bouts of increased energy expenditure per 24 h observing period as compared to LFD controls, each one coinciding with the beginning of either lights off (dark, active) or lights on (light, inactive) phases of the diurnal cycle (Figure 3a). HFDT animals showed a profound increase in energy expenditure, irrespective of the light cycle (Figure 3a).

Respiratory exchange ratio (RER) values varied between 0.76–0.97 for LFD controls and 0.68–0.79 for HFD controls, indicating that mice on a high-fat diet relied more often on fatty acid oxidation as a primary source of energy, as expected (Figure 3b). Triptolide treatments have further decreased the range of RER values for either LFDT (0.67–0.93) or HFDT (0.67–0.77) animals, with mean RER values indicating a small but significant increase in lipid metabolism when compared to respective controls: LFD (0.89 ± 0.05) vs. LFDT (0.84 ± 0.06) and HFD (0.75 ± 0.02) vs. HFDT (0.72 ± 0.02), *p* < 0.001 (Figure 3b). These changes were not associated with either horizontal (Figure 3c) or vertical (Figure 3d) movement activity.

### 3.4. Inflammation of Adipose Tissue

Adipose tissue is a complex environment mainly composed of 59–74% fat cells (adipocytes), 14.8–15.7% adipose-derived stem cells, 0.7–1.8% stromal vascular endothelial cells associated with blood vessels, and several groups of immune cells (1.1–1.8% macrophages, 0.5–3.1% monocytes, 0.4–0.9% CD4+ T cells, 0.5–2.3% CD8+ T cells, and 0.5–5.6% B cells). Omental, pericardial, and epicardial fat tissues contain larger proportions of the immune cells, suggesting that their immune cell archetype is more prone to drive and sustain inflammation as compared to subcutaneous fat [27]. For this reason, we have focused on the expression of the pro-inflammatory gene network in perigonadal visceral fat as a potential source of inflammatory messengers.

**Figure 4:**
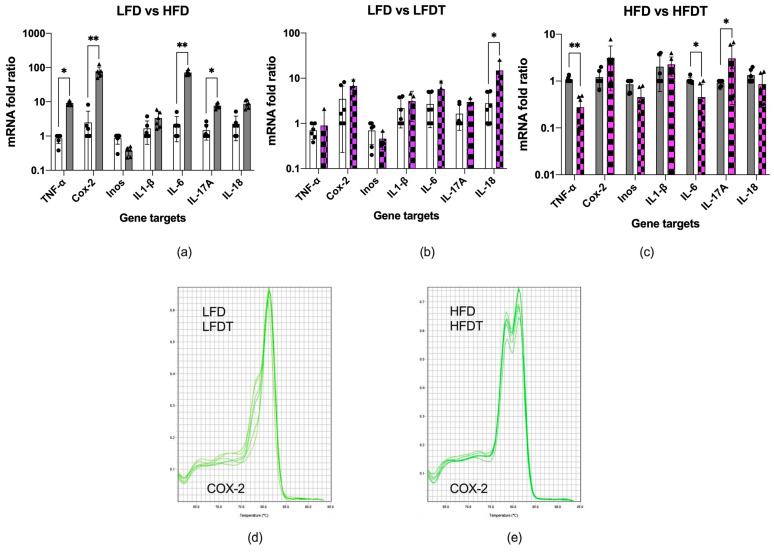
mRNA expression profiles of the inflammatory gene expression networks in the adipose tissue of LFD (dots) and HFD (triangles) animals treated with triptolide. (**a**) Obese HFD animals as compared to LFD controls. (**b**) Lean animals administered with triptolide LFDT as compared to LFD controls. (**c**) Obese animals administered with triptolide HFDT as compared to HFD controls. A putative alternative COX-2 transcript associated with the HFD diet, irrespective of triptolide treatment, was (**d**) absent in LFD and LFDT mice but (**e**) present in HFD and HFDT mice. Fold changes in gene expression are reported as means relative to respective controls (* *p* < 0.05, ** *p* < 0.01).

Obesity resulted in a marked increase in the expression of cyclooxygenase-2 (COX-2) and IL-6 in the adipose tissue of HFD animals, with concurrent moderate increases in TNF-α, IL-17A, and IL-18, while IL-1β changes were minor and inducible nitric oxide synthase (iNOS) expression was reduced (Figure 4a). Despite triptolide being a potent anti-inflammatory agent, IL-18, COX-2, and IL-6 expression were upregulated in the adipose tissues of the LFDT animals (Figure 4b).

In the obese HFDT animals receiving triptolide, we observed a marked suppression of TNF-α and IL-6, but both COX-2 and IL-17A expression remained high (Figure 4c). An unexpected observation occurred when melting dissociation curves were analyzed for COX-2 expression. Consumption of the HFD diet promoted the appearance of a second COX-2 transcript with a different melting point, a likely alternatively spliced mRNA induced by HFD. Triptolide treatment had no effect on the abundance of this transcript (Figure 4d,e).

### 3.5. Analytical Profile of Triptolide and Cytotoxic Adverse Effects

**Figure 5:**
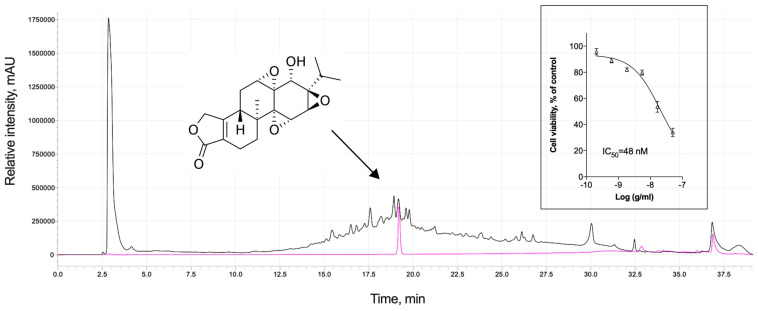
Analytical profile of triptolide, a diterpenoid epoxide (magenta line), in the roots of the thunder god vine *Tripterygium wilfordii* Hook F (black line), and its cytotoxicity profile against RAW 264.7 murine macrophages.

Triptolide accumulates in *Tripterygium wilfordii* Hook F plants and can be readily detected in root tissues (Figure 5). Currently, triptolide has a narrow therapeutic window and induces serious toxicity and adverse effects [28], which explains the very low concentrations used in this study. When tested against RAW 264.7 murine macrophages, cytotoxicity was detected with an IC_50_ value of 48 nM (Figure 5, insert). Our previous pilot studies also observed adverse effects associated with liver toxicity when used in doses exceeding 50 μg/kg in mice [22].

## 4. Discussion

Triptolide is a potent natural pharmacological agent with anti-inflammatory and immunosuppressive properties. As a component of herbal root extract, triptolide was used in traditional Chinese medicine to alleviate rheumatoid arthritis, systemic lupus erythematosus, nephritis, and psoriasis [29]. This was possible in part due to the very low amounts of triptolide found in the plant tissues, ca. 50–70 μg/g dry weight [30].

Triptolide suppresses inflammation by targeting pro-inflammatory gene expression networks modulated by TNF-α and NF-κB signaling pathways [31]. This effect is achieved in part by a broad suppression of gene expression through rapid proteasome-dependent inactivation of RNA polymerase II [20] and the nuclear factor IκBα [21]. As the competing needs of responding to inflammatory stimuli quickly while avoiding overproduction of inflammatory mediators render mRNA translation a critical regulatory checkpoint, many inflammatory mRNAs are short-lived [32]. For this reason, even minor disruptions in mRNA translation have a disproportionately large effect on the abundance of pro-inflammatory cytokines that regulate immune activation. This feature of triptolide was previously applied to mouse models of diabetic nephropathy [33], atherosclerosis [34], nonalcoholic fatty liver [35], and experimental autoimmune encephalomyelitis [36]. Triptolide, its analogs, and herbal preparations containing triptolide have also been tested in clinical settings to alleviate rheumatoid arthritis [37], cancer [38], and other inflammation-related pathologies [29]. However, at higher concentrations, triptolide has a very narrow therapeutic window due to associated serious side effects in the liver and kidneys, which limits its clinical applications [28].

In this study, we took a different approach to addressing this issue. Here, triptolide was used as a pharmacological tool to dissect and decouple the development of obesity and insulin resistance in the polygenic mouse model of high-fat diet-induced obesity by selectively targeting the most sensitive pro-inflammatory mRNA targets. This was achieved by microdosing triptolide at 0.1 μg/g food (estimated intake of up to 10 μg/kg/d), which is 10–50× lower than a typical dosing range of 100–500 μg/kg/d used in the previous animal studies. At this level, triptolide administration has not prevented the development of obesity and caused no significant changes in body composition or energy expenditure. Instead, the animals treated with triptolide have been presented with a healthier obese phenotype characterized by a 15–36% improvement in insulin resistance and a 38–49% improvement in blood glucose levels. This phenotype mimics the metabolically healthy overweight/obese phenotype described in humans earlier [7]. Subjects with such a metabolically healthy obese phenotype are expected to have superior levels of clinical risk factors compared to unhealthy individuals, as shown in the NHANES-III study [39].

Increased fat associated with obesity leads to a pro-inflammatory pathological state of the adipose tissue characterized by the accumulation of adipose tissue macrophages skewed towards the M1 profile [10]. They are functionally similar to Kupffer macrophages in the liver, transient muscle macrophages, alveolar macrophages in the lungs, and microglia in the brain. These cells express the short-lived core cluster of effector cytokines and chemokines that simultaneously drive the pro-inflammatory responses and the development of insulin resistance to ensure the availability of energy substrates for the expanding immune and adipose tissues [40]. The gene network centers on TNF-α that interacts with the TNF receptor superfamily to activate NF-κB and promote temporal modulation of immune responses and metabolism via IL-1β/IL-6/IL-17 and IL-18/INF-γ/MCP-1 signaling. Downstream, cyclooxygenases such as COX-2 and nitric oxide synthases such as iNOS drive the production of prostaglandin E_2_ and NO, respectively. This network features several remarkable overlaps, both with the cytokine storm observed in viral infections and exacerbated in obesity [41], as well as insulin-resistant metabolic conditions [42].

While obesity was associated with upregulation of many effector molecules, TNF-α, COX-2 and IL-6 expression showed the strongest responses. Microdosing with triptolide returned TNF-α expression to basal levels but further upregulated COX-2 and IL-17A expression, suggesting that TNF-α is a critical detrimental link between inflammation and the development of insulin resistance, while adipose tissue COX-2 and IL-17A may offer some level of protection. However, previous clinical studies suggested that targeting TNF-α [43] or the TNF-α receptor [44] alone was not sufficient to improve glycemic control in diabetes. The newly emerging evidence also supported our conclusions, as higher levels of adipose tissue COX-2 expression were found to improve adipose tissue dysfunction [45], while increased IL-17 (IL-17A)-mediated metabolic regulation was beneficial for repair of the barrier tissues and glucose homeostasis [46].

Moreover, the mRNA expression analysis indicated the presence of an alternative COX-2 transcript variant in obese animals. The significance of this observation remains unknown, but a recent report of the changes in the most common COX2.1 isoform expression in favor of the COX2.2 variant associated with chronic rhinosinusitis [47] suggests a possibility of alterations in the transformation of the arachidonic acid metabolism that may be associated with high fat diet.

## 5. Conclusions

Dysregulation of metabolism and inflammation is a common feature of obesity, and the high metabolic demands of the activated immune component may be partially responsible for increased activity of the anabolic glycolysis and pentose phosphate pathways at the expense of the tricarboxylic acid cycle and oxidative phosphorylation [40]. To achieve this shift, insulin resistance is set and maintained in key immune and metabolic tissues. In healthy, infected, or wounded states, this shift is transient and resolves with time, as homeostasis ensures.Chronic metabolic states do not achieve resolution, and microdosing with triptolide can therefore be used as a pharmacological tool to dissect the molecular mechanisms that support the co-development of obesity and insulin resistance in these situations. Specific future focus should be maintained not only on suppression of TNF-α signaling as a critical target in the onset of insulin resistance, but also on the putative protective roles of the COX-2-mediated or IL-17A-mediated pathways in metabolic disorders.

## Data Availability

The data are contained within the article.
